# The Friendship Bench as a brief psychological intervention with peer support in rural Zimbabwean women: a mixed methods pilot evaluation

**DOI:** 10.1017/gmh.2021.32

**Published:** 2021-08-26

**Authors:** Shamiso Fernando, Tim Brown, Kavita Datta, Dzivaidzo Chidhanguro, Naume V. Tavengwa, Jaya Chandna, Epiphania Munetsi, Lloyd Dzapasi, Chandiwana Nyachowe, Batsirai Mutasa, Bernard Chasekwa, Robert Ntozini, Dixon Chibanda, Andrew J. Prendergast

**Affiliations:** 1 Zvitambo Institute for Maternal and Child Health Research, Harare, Zimbabwe; 2 Queen Mary University of London, London, UK; 3 African Mental Health Research Initiative (AMARI), Harare, Zimbabwe; 4 Ministry of Health and Child Care, Harare, Zimbabwe

**Keywords:** Depression, problem-solving therapy, Africa, task sharing, community health workers

## Abstract

**Background:**

There is a large treatment gap for common mental disorders in rural areas of low-income countries. We tested the Friendship Bench as a brief psychological intervention delivered by village health workers (VHWs) in rural Zimbabwe.

**Methods:**

Rural women identified with depression in a previous trial received weekly home-based problem-solving therapy from VHWs for 6 weeks, and joined a peer-support group. Depression was assessed using the Edinburgh Postnatal Depression Scale (EPDS) and Shona Symptom Questionnaire (SSQ). Acceptability was explored through in-depth interviews and focus group discussions. The proportion of women with depression pre- and post-intervention was compared using McNemar's test.

**Results:**

Ten VHWs delivered problem-solving therapy to 27 women of mean age 33 years; 25 completed six sessions. Women valued an established and trustful relationship with their VHW, which ensured confidentiality and prevented gossip, and reported finding individual problem-solving therapy beneficial. Peer-support meetings provided space to share problems, solutions and skills. The proportion of women with depression or suicidal ideation on the EPDS declined from 68% to 12% [difference 56% (95% confidence interval (CI) 27.0–85.0); *p* = 0.001], and the proportion scoring high (>7) on the SSQ declined from 52% to 4% [difference 48% (95% CI 24.4–71.6); *p* < 0.001] after the 6-week intervention.

**Conclusion:**

VHW-delivered problem-solving therapy and peer-support was acceptable and showed promising results in this pilot evaluation, leading to quantitative and qualitative improvements in mental health among rural Zimbabwean women. Scale-up of the Friendship Bench in rural areas would help close the treatment gap for common mental disorders.

## Introduction

Depressive disorders are among the leading global causes of disability-adjusted life years (GBD 2019 Diseases and Injuries Collaborators, 2020), with a disproportionate burden in low- and middle-income countries (Walker *et al*., 2015). The age of onset is frequently in early adulthood and prevalence in women tends to be 2–3-fold higher than that in men (Patel *et al*., 2009). Among reproductive-age women, depression is a risk factor for low birthweight (Grote *et al*., 2010), and poor mental health compromises caregiving (Field, 2010), feeding practices (Dennis and McQueen, 2009) and parenting behaviours (Lovejoy *et al*., 2000). Children of mothers with depression have higher odds of undernutrition (Surkan *et al*., 2011), and impairments in early child development and behaviour (Britto *et al*., 2017). For women themselves, common mental disorders cause considerable disability, and incur substantial social and economic costs (Patel *et al*., 2009).

Psychological interventions for common mental disorders delivered through task-sharing are efficacious and cost-effective in low-income countries (Patel *et al*., 2009) but there is a large treatment gap, particularly in rural areas (Chibanda, 2017). The Friendship Bench is a brief psychological intervention delivered by trained lay health workers through individual problem-solving therapy (Chibanda *et al*., 2016). In Zimbabwe, depression is encapsulated in the local concept of ‘thinking too much’ (*kufungisia* in Shona) (Patel *et al*., 2001), and problem-solving therapy has been shown to be effective at managing *kufungisisa*. In a cluster-randomised trial in urban Zimbabwe, participants receiving the intervention had a significant reduction in common mental disorders at 6 months and lower risk of depression symptoms compared to enhanced usual care [13.7% *v*. 49.9%; adjusted risk ratio 0.28; 95% confidence interval (CI) 0.22–0.34] (Chibanda *et al*., 2016). The Friendship Bench has therefore been scaled up to over 100 primary care clinics in urban Zimbabwe, narrowing the treatment gap (Chibanda, 2017).

The Friendship Bench has not been evaluated outside urban centres, although two-thirds of the Zimbabwean population resides in rural areas. Undernutrition is more common in rural compared to urban settings (Black *et al*., 2013), so an intervention aimed at promoting child growth and development through improved maternal mental health needs to work in this context. The Friendship Bench could easily be adapted for rural households, particularly if delivered by community health workers. We therefore set out to evaluate whether the Friendship Bench could be delivered to reproductive-age women in a rural Zimbabwean district.

## Methods

### Study setting

This study was conducted in rural Shurugwi, a subsistence farming district in central Zimbabwe with high rates of food insecurity, 15% HIV prevalence and 35% child stunting prevalence (Humphrey *et al*., 2019). We previously conducted the Sanitation Hygiene Infant Nutrition Efficacy (SHINE) trial in the same district between 2012 and 2017 (Humphrey *et al*., 2019). SHINE enrolled a cohort of pregnant women and evaluated the impact of improved infant nutrition and/or improved water, sanitation and hygiene on child growth and development. In SHINE, 343/3941 (8.7%) of women had clinically significant depression during pregnancy (Tome *et al*., 2020) using locally validated tools (Chibanda *et al*., 2010), but no community-based treatment interventions were available.

In the SHINE trial, all behaviour-change communication was delivered by village health workers (VHWs). The general requirement for VHWs in Zimbabwe is women or men above the age of 25 years, preferably well established in that community, who can read and write, are respected by the community, and are chosen by the community where they reside. The aim of the current study was to evaluate whether the Friendship Bench intervention could be successfully delivered to rural reproductive-age women by VHWs. We conducted focus-group discussions with VHWs delivering the intervention, in-depth interviews with women receiving the intervention, and compared pre- and post-intervention mental health scores using several validated tools (Chibanda *et al*., 2010). We hypothesised that the Friendship Bench would be acceptable to women and would improve maternal mental health and responsive caregiving.

### Theory of change

An initial meeting was held with district stakeholders to develop a theory of change road map, which systematically mapped out the steps that would lead to achieving the study aims (Appendix 1). Community members and clinic staff in the catchment areas were sensitised about the study. Problem-solving therapy teaches a participant to use a step-by-step approach to solve life challenges, which involves identifying and defining a problem, attempting to understand the problem and then generating and implementing solutions to address the problem (Malouff *et al*., 2007). The Friendship Bench approach is based on indigenous concepts of problem-solving therapy: opening the mind (Kuvhura pfungwa), uplifting (kusimudzira), strengthening and strengthening further (kusimbisa and kusimbisisa), respectively. Using terms locally conceived and developed by lay health workers to describe components and processes of problem-solving therapy contribute to the acceptability and continued use of the Friendship Bench in primary care facilities (Chibanda *et al*., 2017).

### VHW training

VHWs received 5 days of residential training by a team comprising a research nurse, District Mental Health Nurse, District VHW Trainer and five Friendship Bench trainers; content included teaching on depression, training in counselling skills, and role plays. VHWs were encouraged to practice these new skills, and then had 2 days of refresher training. VHWs were grouped into three clusters to receive ongoing mentorship, support and supervision at their local clinics. VHWs were directly supported by a Research Nurse Supervisor working full-time on the study; she would provide supportive supervision by sitting in on sessions or practising role plays ahead of a session. The Research Nurse Supervisor did not have mental health expertise, but had access to the District Mental Health Nurse in Shurugwi to provide advice on management or onwards referral of difficult cases, and by the Friendship Bench team in Harare who provided remote support.

### Study participants

**Fig. 1.**
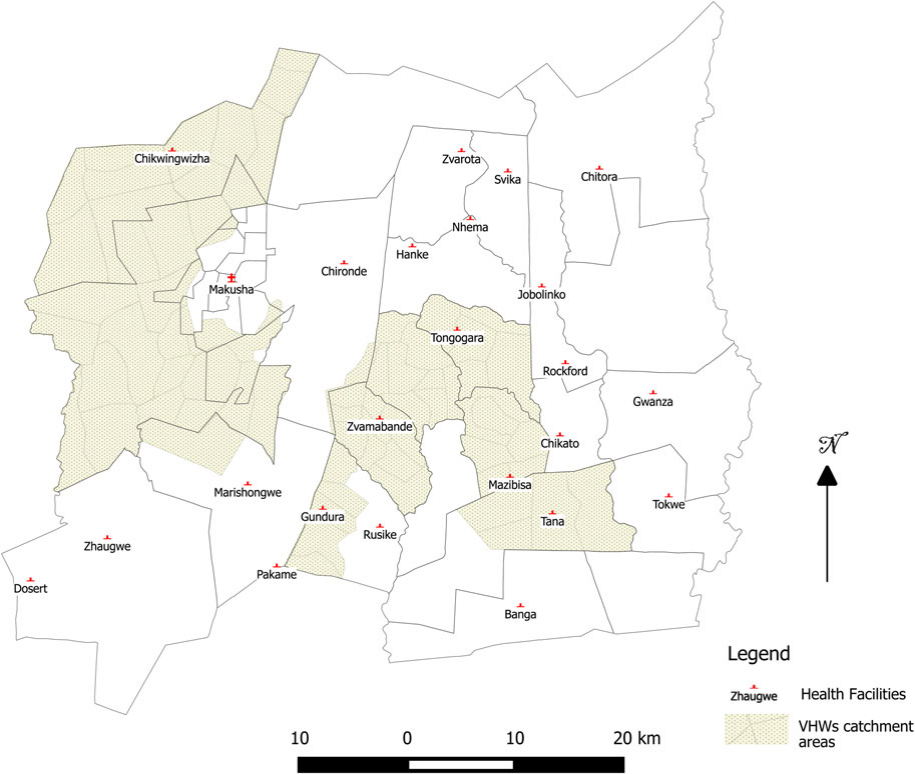
Map of the study area.

We enrolled women to the current study who had previously participated in the SHINE trial and had been identified with depression. Women with symptoms of depression were identified from the SHINE trial database. Depression was assessed in SHINE during pregnancy, then at 1, 6, 12 and 24 months postpartum using a Shona version of the Edinburgh Postnatal Depression Scale (EPDS), which has previously been validated in urban Zimbabwe (Chibanda *et al*., 2010). Women scoring ⩾12 on the EPDS, or answering ‘Yes, quite often’ or ‘Sometimes’ to the question ‘Has the thought of harming yourself occurred to you?’ at 24 months and/or an earlier timepoint were categorised with clinically significant depression (Tome *et al*., 2020). From this group of 272 women, 30 were purposively sampled for the current study if they resided in the catchment area of six rural health centres (Gundura, Makusha, Mazivisa, Tana, Tongogara and Zvamabande; Fig. 1). This study area was chosen (i) to be accessible from Shurugwi town, where the study team was based; (ii) to ensure that women were sufficiently close to establish peer-support groups and (iii) to be near major clinics, which provided a base for VHW meetings.

### Enrolment and baseline assessment

Eligible women were approached by their VHW to provide further information about the study. Women who expressed interest were visited by a research nurse to seek written informed consent. Following enrolment, maternal mental health was assessed using the EPDS (Chibanda *et al*., 2010), Shona Symptom Questionnaire (SSQ) (Patel *et al*., 1997), WHO Disability Assessment Schedule (WHODAS) and Generalised Anxiety Disorder Assessment (GAD-7) (Spitzer *et al*., 2006) (see online Supplementary material). The EPDS is a widely used 10-item self-report questionnaire that was originally developed to identify postnatal depression (Cox *et al*., 1987), but has been validated as a screening tool for depression in women with older children (Cox *et al*., 1996). Each item is scored from 0 to 3, leading to a total score between 0 and 30, with higher scores indicating more severe depressive symptoms. In Zimbabwe, a cut-off score of 11/12 provided the optimum sensitivity (88%), specificity (87%), positive predictive value (74%) and negative predictive value (94%), using the DSM-IV as a gold standard (Chibanda *et al*., 2010). The SSQ is an indigenous instrument developed in Zimbabwe for detection of common mental disorders (Patel *et al*., 1997). It is a 14-item questionnaire soliciting symptoms over the past 7 days, which is widely used as a culturally relevant tool for detection of common mental disorders in sub-Saharan Africa; a score of >7 positive items (out of a maximum score of 14) yields a sensitivity of 67% and specificity of 83% (Patel *et al*., 1997). The WHODAS is a generic assessment instrument covering domains of cognition, mobility, self-care, interacting with other people, life activities, and participation, which produces standardised disability levels applicable across diseases and cultures. Each of 12 items is scored as ‘none’, ‘mild’, ‘moderate’, ‘severe’ or ‘extreme’; higher scores indicate more severe disability. The GAD-7 is a 7-item self-reported assessment scale for anxiety (Spitzer *et al*., 2006), which has been validated in sub-Saharan Africa (Chibanda *et al*., 2016). Each item is scored 0–3 and total GAD-7 scores range from 0 to 21, with higher scores reflecting greater severity of anxiety; cut-off scores of 5, 10 and 15 identify mild, moderate and severe anxiety symptoms, respectively. Responsive caregiving was assessed using the Observation of Maternal–Child Interaction (OMCI) tool, which was developed and validated in rural Pakistan to measure mother–child interactions during a 5 min observation. The mother and child are given a story book that was developed to reflect the local context. The assessor asks the mother and child to play as they normally would and sits to the side, marking the interaction. The scoring is based on a maternal and infant interaction framework (Landry *et al*., 2006) and comprises 12 maternal behaviour items, 6 child observation items and 1 mutual enjoyment item. Maternal behaviours assessed include affect, touch, verbal statements and language stimulation; child behaviours assessed include affect, communication and attention. Each item is scored on a scale of 0–3 (0 = never observed, 3 = observed over 5 times) with a maximum possible score of 57. A higher score indicates a more responsive interaction (Rasheed and Yousafzai, 2015). A research nurse was trained in the OMCI tool by a child psychologist (JC); assessments were standardised through concurrent marking of ten OMCI assessments, with >95% average agreement between scores.

### Friendship Bench intervention

VHWs conducted weekly home visits for 6 weeks, using a structured approach to identify problems and generate solutions. VHWs were trained to identify ‘red flags’ indicating clinically significant depression or suicidal ideation; these women were referred to local clinics or discussed with the district mental health nurse. After the third session, women were invited to join a peer-support group known as Circle Kubatana Tose (‘holding hands together’; CKT) at their local clinic; women therefore joined the groups at different times. CKT groups bring women together to share and solve problems, and to initiate income-generating activities. The first six sessions included a VHW facilitator, refreshments and transport reimbursement; subsequently, women were encouraged to maintain peer-led CKT groups and income-generating activities themselves, and to open up the groups to non-study participants if they wished.

### Quantitative evaluation

After six sessions of problem-solving therapy, women received an endline visit by a research nurse, who repeated the mental health and OMCI assessments. Quantitative data were collected onto paper case report forms, checked by a nurse supervisor, and entered onto a study database.

### Qualitative evaluation

Semi-structured interviews were conducted in Shona by a trained researcher with 10 enrolled women. In order to explore a range of experiences, women were purposefully sampled based upon their household characteristics (e.g. family size and household structure) as well as on VHW judgements about their potential as information-rich cases (Patton, 2002). The sample size allowed for credible and meaningful insights given other characteristics the women shared (e.g. ethnicity, gender, poverty and rural dwelling) and their participation, sometimes together, in the CKT groups. Interviews lasted 30–60 min and were digitally recorded, transcribed verbatim and translated into English. All VHWs delivering the intervention were invited to a 2-h focus group discussion in Shona. Both the focus group and semi-structured interviews began with an ‘ice-breaker’ exercise which involved participants hearing a fictional vignette, then describing the health problems faced (including maternal depression), the potential causes and their impact on the woman's familial and wider social interactions and relationships. Subsequently, an interview schedule was used to prompt discussion of symptoms and causes of maternal depression; prevalence of, and community response to, maternal depression and experience of the Friendship Bench intervention.

### Analysis

Pre- and post-intervention scores were compared using paired *t* tests or Wilcoxon-signed rank tests depending on distributions. Proportions were compared using McNemar's test. All quantitative analyses used Stata version 14 (StataCorp LLC, College Station, TX). Interviews and focus group transcripts were coded using both deductive and inductive approaches based upon the framework method often employed in multidisciplinary health-related research (Gale *et al*., 2013). Deductive codes were developed relating to the primary goal of evaluating the Friendship Bench intervention, its mode of delivery and perceptions of its effectiveness. Inductive codes were developed by reading a randomly selected transcript line-by-line and applying codes to more text than in the deductive approach to broaden the range of themes. Following discussion, both the deductive and inductive codes were refined and applied to a further sample transcript and the process repeated until the codes were thorough and could be accurately used for analysis (Table 1). Findings from the qualitative research are presented using anonymised case-codes applied to the individual participants rather than pseudonyms (e.g. 70 009); the prefix VHW is applied ahead of an individual number applied to the VHWs who engaged in the focus group discussion (e.g. VHW-01).

**Table 1.** Coding frame and themes

Main codes	Sub-codes and themes
Maternal depression	Signs and symptoms ‘Thinking too much’ Embodiment Behaviour change Emotions Knowledge Understanding/beliefs Sources of knowledge Personal experience Causes of depression Domestic life Childbirth/pregnancy Poverty/work Illness Children Impact of maternal depression Support provided
Intervention experience	Selection Influence of past interventions Emotional response Anxieties Participation Facilitators Mode of intervention Benefits/challenges Role of gossip VHW role

### Approvals

The study was approved by the Medical Research Council of Zimbabwe and the institutional review board of the Johns Hopkins Bloomberg School of Public Health.

## Results

### Study flow and baseline characteristics

Between 11 and 24 September 2018, 30 women were approached to join the study and 27 enrolled; two women declined and one had relocated since the end of the SHINE trial. Women enrolled into the study mean (s.d.) 4.2 (0.7) years after delivery of their infant, and 2.7 (0.7) years after the end of the SHINE trial. Women were mean 33.0 (s.d. 6.4) years old, had median 10 (IQR 7, 11) years of education, 93% were married or living with a partner and 52% were of the Apostolic religion (Table 2). Compared to SHINE women who were not enrolled in this study, enrolled women were broadly similar in baseline demographic characteristics, although they were significantly more likely to be in the bottom wealth quintile, and less likely to be in the highest wealth quintile (online Supplementary Table S1).

**Table 2.** Baseline characteristics of enrolled women

Maternal factors^a^	*N* = 27
Age, years; mean (s.d.)	33.0 (6.4)
Years since exiting SHINE; mean (s.d.)	2.7 (0.7)
Height, cm; mean (s.d.)	161.0 (6.0)
Married, % (*N*)	92.6 (25)
Completed schooling, years; median (IQR)	10 (7, 11)
Employed, % (*N*)	0.0 (0)
Religion, % (*N*)	
Apostolic	51.9 (14)
Other Christian	29.6 (8)
Other religion	18.5 (5)
HIV-positive, % (*N*)	29.6 (8)
Household factors^a^	
Household size; median (IQR)	5 (3, 6)
Electricity, % (*N*)	3.9 (1)
Drinking water from improved source, % (*N*)	69.2 (18)
Wealth quintile, % (*N*)	
Lowest	42.3 (11)
Lower middle	23.1 (6)
Middle	7.7 (2)
Upper middle	19.2 (5)
Highest	7.7 (2)

a Maternal age was calculated at the time of the baseline assessment for the current study. All other maternal and household factors were assessed at the time of enrolment to the SHINE trial.

At baseline, 17/27 (63%) had clinically significant depression scores as measured by the EPDS, and 13/27 (48%) scored >7 on the SSQ. Of the 27 enrolled women, one reported no problems and one moved away after the first two sessions; 25 women therefore completed the programme of six problem-solving sessions over 6 weeks.

### Delivery of the Friendship Bench by VHWs

Ten VHW (eight female, two male) delivered the Friendship Bench intervention, with a median of 3 (range 2–4) participants each. VHW had a median age of 46 years (range 34–55 years), 8 (80%) had completed secondary education and they had median 14.5 years of service as a VHW (range 9–24 years). Eight VHWs required no additional support during delivery of the problem-solving therapy sessions; two required extra support from their nurse supervisor to gain a deeper understanding of the problem the participant had identified, and to help her generate solutions. Nine VHWs attended the focus group discussion, median 12 days (range 8–19 days) after delivering the final problem-solving session.

A defining feature of the Friendship Bench is the emphasis on problem solving (Chibanda *et al*., 2016). As expected, this element was identified as important by VHWs. When discussing their experiences, VHWs identified their role as helping to ‘open up the minds’ of the women to encourage them to identify their problems and possible solutions (VHW-02 and VHW-09). The support of more experienced mental health professionals was important when VHWs encountered ‘red flags’, which were identified in three women (e.g. cases where women discussed suicidal ideation).

VHWs revealed the difficulties they faced encouraging some participants to ‘open up’. As one VHW remarked, ‘it was not easy for anyone to openly discuss their problems or secrets’ (VHW-06). VHWs recounted the importance of the training they received and their own anxieties about addressing this sensitive topic, feeling that they had ‘very little knowledge’ (VHW-01) or were ‘not yet confident’ (VHW-03) at the beginning. Some mentioned they lacked confidence in delivering specific elements of the intervention; for example, the role plays that were employed to promote awareness of maternal depression. However, the closely entwined issues of confidentiality and gossip caused the greatest concern: ‘I was worried that she [female participant] would think that ‘if I tell the VHW my problems, I will end up hearing about them somewhere else’’ (VHW-01). Others agreed, with one VHW noting that it was not easy because ‘gossips spread in the village’ and that it ‘does not come from one person’, which leaves people afraid to talk (VHW-06).

### Acceptability of the Friendship Bench by participants

Interviews with 10 participants were conducted median 13 days (range 7–19 days) after the last problem-solving session. Interviews suggested that many of the women responded well to the intervention, with one commenting that she ‘did not see anything that did not work well in the Friendship Bench’ (70 009). This sense of the intervention's acceptability was made apparent through the women's discussion of the ways in which it supported them to deal more effectively with problems that they were experiencing. However, its acceptability might also be judged by the request that several of the participants made about keeping the intervention going: ‘I just want to say keep the Friendship Bench going because you are helping a lot of women’ (70 001), ‘Ahh, I do not have any questions but I would just like to say if it was possible, it would be nice to keep having sessions’ (70 008).

The problem-solving approach was not regarded as entirely novel; for example, women discussed the similarities with, as well as differences from, other forms of community-based support, including that provided by religious leaders. However, it was identified as being of particular benefit by all participants. As one described, ‘[w]e used to have problems that were so bad that even if we prayed at church or tried to say anything, they were just difficult to solve. We had problems that were really bad, no matter how much you prayed or what you said’ (70 009). This participant described how, starting with the biggest first, she was able to get the problems ‘off her chest’ and that she and the other women ‘realised that there is no problem without a solution’.

Women faced multiple, overlapping individual, familial and wider societal problems. Under such circumstances, the process of ‘opening up’ to the VHWs often took time; as one described, the first counselling session went well, but they were ‘not interested’ and only participated for the ‘sake of it’. For this woman, the focus on problem-solving financial difficulties prompted her to engage more fully: ‘[w]hat made me open up my mind was that my VHW asked if I felt able to start a business … and he said I could start, even with whatever amount of money that I got’ (70 015).

Interviews revealed that having contextually relevant and trustworthy health workers was an important element for women (Chibanda *et al*., 2016). VHWs were already known to participants through their involvement in the SHINE trial (Humphrey *et al*., 2019). This prior interaction helped to establish a relationship of mutual respect and trust:

‘… what makes me trust her [the VHW] is that I did not just start working with her on these issues only, ah, I trust her, there are other things that I have previously worked on with her, some of the things are those that were not meant to be known by other people and she keeps it that way. Just as we used to during SHINE. She used to tell me that even if you get tested, no one will know your results. Or if we test your child and find out s/he isn't well no one will know. So that's how it always was. … That's why I kept on trusting her.’ (70 008)

This combination of an established and trustful relationship with the VHWs was established through the VHWs' discretion with regards to sharing confidential information with others, and through their willingness to engage participants in discussion of their working practices:

“Interviewer: What is it that made you trust her? She lives in the same village as you and probably knows about your life and talks to many other people?

Participant: Ah, I noticed that if you tell her anything, as an adult she knew that…she never talks to people about confidential issues. I noted that she doesn't share with anyone else.

Interviewer: In what way did they provide assurance?

Participant: She also told me that she is not supposed to go around telling people what she would have discussed with a participant. She said she is not supposed to do that, she is supposed to go home and pack her books and continue with her work. (70 004)

This emphasis upon trust was closely associated with participant concerns over gossip, which played an important role in their rural lives and could act as a barrier to participation in an intervention that required sharing intimate details about their mental ill-health: ‘They say “you see this one, she did this and that” like what happened to me yesterday when I was at our rural home’ (70 022). Here, the participant starts by discussing gossip in quite general terms before recounting an experience related to her embodiment of *Kufungisisa* (‘thinking too much’): ‘Yes, you can hear them talking about you as you will be walking around, like what happened to me this other year, when I was still living here in Shurugwi. This woman once asked me in the bathroom that “ah you! are you ill?” This was because she had seen how skinny I was’. In this instance, the fear of generating gossip about her appearance, especially because of the association between weight-loss and HIV/AIDS, prevented the participant from returning to her local bath-house. The participant also revealed a more generalised anxiety over the potential to become the focus for gossip: ‘they will be looking at you losing weight and conclude that you are thinking too much or not feeling well’. As this suggests, being able to trust VHWs is vital to women's participation in such an environment.

### Peer-to-peer support groups

Three CKT groups were established, comprising 6, 9 and 10 study participants, respectively; each group met weekly. Income-generating activities included making mats, bags, hats, cooking sticks and hoe handles for sale; buying and selling vegetables; and raising chickens.

CKT groups extended the support provided through the individualised and personalised problem-solving sessions, and the benefits were apparent to VHWs and participants. As one VHW noted, the CKT meetings built upon the support they were providing through the one-to-one sessions: ‘when we went to meet with the other women, we noted that people have different kinds of problems’ (VHW-07). CKT meetings provided space to share problems, and opportunities to share solutions:

‘if you look at the sessions where we discuss with the mothers, if she chooses a solution that works well for her, it is another method that is going to help in the CKT sessions, for example if she says, ‘I had trouble securing school fees for my children’ there may also be 2 or 3 other members who are also struggling with school fees so if they find out how she managed to raise school fees, it will help other 2 or 3 of them to also get school fees’. (VHW-06)

The challenges of living in extreme poverty and the inability to generate income to meet every day needs was of central concern for women (ZimVac, 2020); this situation is exacerbated by gender-based power relations that often leave women reliant on absent husbands for support. Here, the capacity of the CKT meetings to move beyond individuated solutions to collective ones appeared especially important. For example, many women highlighted the skills they were taught and subsequently shared in knitting items for sale using recycled plastics, paper, discarded clothes or other materials. Although this skill-sharing raised challenges (e.g. the need to source materials) the participants also reflected on the importance of these shared capacity-building activities: ‘We teach each other. Each individual shares what they know. Sometimes before we hoard, someone shares with us what she knows and we do that … When someone shares what they know you learn from them’ (70 009).

A range of income-generating practices were developed through the CKT groups. Women shared examples of working together to increase their capacity to purchase goods for onwards sale (e.g. chickens, fish and maize) and drawing on the knowledge of their newfound skills to identify the value of unwanted household items:

‘[I]f I do not have money to go and buy in town I can wash my old or torn up clothes and start selling them to people who may need door mats or anywhere else, and I can get $2 or a dollar, and I would have made my money to take my maize to the grinding mill’. (70 009)

Although there is wider benefit to these income-generating schemes, their relevance to women's experience of depression was reflected on throughout the interviews. In the case of this participant, the activities helped improve her experience of depression: ‘[d]epression? It helped in that I no longer think too much’. Most women identified poverty as a significant cause of depression and highlighted the importance of the CKT meetings in helping them to alleviate their symptoms.

Importantly, it was not only women's ability to share problems and solutions with others that was of value to them but also that the CKT meetings provided them with the voice to do so. As one woman reflected, ‘I used to pinch myself when I no longer felt as though I still existed’, noting further that at these moments she felt ‘dead’ and questioned ‘what can I say in front of people?’. The CKT meetings appeared to support women and value their contributions: ‘I never used to be able to open my mouth to talk at a meeting with other people’. Her experience of the CKT meetings altered this sense of self: ‘I would say to myself, “ah, this is possible? I can also say something good that other people can listen to?” I really felt like a person’ (70 009).

### Pre- and post-intervention mental health scores

The 25 women who completed all six problem-solving sessions were evaluated at endline, median 5 days (IQR: 4, 6) after the final session. There were significant reductions in depression and anxiety scores following the intervention. The SSQ score declined from mean 7.2 (s.d. 3.4) at baseline to 1.6 (2.6) post-intervention [mean difference 5.7 (95% CI 4.1–7.2); paired *t* test *p* < 0.001], demonstrating a reduction in symptoms of common mental disorders. Using the SSQ, the proportion of women identified with clinically significant scores (SSQ > 7) at endline was 4%, compared to 52% at baseline; proportion difference 48% (95% CI 24.4–71.6); *p* < 0.001. The EPDS declined from mean 22.4 (s.d. 5.9) to 13.3 (5.0) [mean difference 9.1 (95% CI 6.0–12.1); paired *t* test *p* < 0.001], which reflects a clinically significant decline in depression scores (Matthey, 2004). Using the EPDS, the proportion of women with clinically significant depression scores (EPDS ⩾ 12) or suicidal ideation declined from 68% to 12%; proportion difference 56% (95% CI 27.0–85.0); *p* = 0.001.

The WHODAS score declined from median 14.6 (IQR: 6.3, 27.1) at baseline to 0.0 (0.0, 4.2) post-intervention (Wilcoxon-signed rank test *Z* = 3.49, *p* < 0.001), demonstrating a reduction in self-reported disability among women following the intervention. The GAD-7 score declined from median 7.0 (IQR: 1.0, 10.0) to 0.0 (0.0, 0.0); *Z* = 3.73, *p* < 0.001, reflection a reduction in self-reported anxiety. The proportion of women with clinically significant anxiety disorder scores (GAD-7 > 5) declined from 64% to 16%; proportion difference 48% (95% CI 24.4–71.6); *p* < 0.001.

The OMCI was undertaken with children aged mean (s.d.) 4.25 (0.74) years. The OMCI score declined between baseline and endline, from 31.5 (s.d. 4.7) to 29.0 (5.1); mean difference 2.6 (95% CI 0.2–4.9); paired *t* test *p* = 0.035, indicating reduced maternal–child interaction following the intervention. This difference was driven by a decline in the maternal component of the score, from 21.1 (s.d. 3.0) at baseline to 18.6 (2.9) at endline (mean difference 2.4, 95% CI 0.6–4.3; paired *t* test *p* = 0.011), but no significant difference in the child score [baseline 10.4 (s.d. 3.1) *v.* endline 10.3 (3.1); mean difference 0.1 (95% CI−1.1 to 1.4); paired *t* test *p* = 0.84].

### Sustainability of the Friendship Bench

Although it was beyond the scope of this study to explore long-term sustainability, participants in interviews often associated the benefits of the Friendship Bench intervention with their improved capacities to generate money. It was often this that helped to ‘relax their minds’. By December 2019, 1 year after the study ended, 13/25 (52%) women were still actively engaged in CKT groups. Four groups (67%) had maintained or expanded their income-generating projects, and several groups had expanded to involve other community members. Among the 48% of women who were no longer engaged in CKT groups, the reasons cited were loss of ongoing VHW support after the study ended; lack of motivation for mothers in the form of refreshments (which they could not afford to supply); and challenges selling items that they produced due to economic hardship in the local community.

## Discussion

In this pilot study, we evaluated whether the Friendship Bench, which has been scaled up as an evidence-based intervention in urban Zimbabwe (Chibanda *et al*., 2016; Chibanda, 2017), could be successfully delivered by VHWs in rural Zimbabwe, where the majority of people live. Our mixed methods evaluation demonstrated that the intervention was highly valued by women and by VHWs, and that the combination of individualised therapy and peer-to-peer support led to quantitative and qualitative improvements in mental health, problem solving and income generation, despite multifaceted challenges in their rural lives. After six intervention sessions, the proportion of women with clinically significant depression scores on the EPDS declined from 68% to 12%, which is similar to prior findings from urban settings (Chibanda *et al*., 2016; Munetsi *et al*., 2018), showing that a home-based approach to delivering the intervention in rural areas by VHWs is highly promising. One year after all study inputs ended, half of the support groups were still running, several had expanded in size, and the majority continued income-generating activities. Collectively, our findings demonstrate the enormous potential of the Friendship Bench in rural areas to help close the treatment gap for common mental disorders (Chibanda, 2017).

Qualitative findings highlighted the central importance of trust between women and their VHW in their willingness to discuss sensitive and personal issues. Participants also placed much value on the CKT peer-support meetings. Although these have been a feature of the Friendship Bench for over 10 years, this is the first time this intervention component has been specifically evaluated. To an extent, the value of CKT groups can be understood in monetary terms, as women used the networks they established to extend their income-generating opportunities. The emphasis on addressing poverty- and gender-related issues through the CKT groups highlights the well-established causal relationship between poverty and common mental disorders (Lund *et al*., 2010). As an anti-poverty initiative, CKT groups further reduce depression and sustain improvements gained through the initial problem-solving therapy sessions. However, the value of the CKT meetings extended beyond financial benefits. Recognising that many women in this study live in remote rural areas and in challenging circumstances, CKT groups were identified as spaces that allowed women to share with each other, engage in problem-solving activities and build confidence and a stronger sense of self, including the contributions they could make. Such an understanding fits well with more nuanced understanding of gender and the lived experience of women in rural Zimbabwe (Bhatasara and Chiweshe, 2017).

Our initial motivation for evaluating the Friendship Bench in rural Zimbabwe stemmed from the SHINE trial (Humphrey *et al*., 2019), which identified a substantial burden of depression in pregnancy and a lack of locally available interventions (Tome *et al*., 2020). Previous studies have shown that maternal depression contributes to child undernutrition (Surkan *et al*., 2011), and the UNICEF framework highlights the central role of maternal caregiving in promoting healthy growth and development. In SHINE, we found that the randomised infant feeding intervention ameliorated the adverse impact of depression on child growth (Tome *et al*., 2020). However, we did not explicitly include an intervention to tackle maternal depression. It is therefore encouraging that problem-solving therapy and peer-support groups delivered by VHWs are an acceptable and promising strategy for improving mental health, since this may have benefits for the child.

The WHO/UNICEF Nurturing Care Framework specifically recognises the importance of responsive caregiving for child development (World Health Organization, United Nations Children's Fund, World Bank Group, 2018). We therefore included an assessment of maternal–child interaction (Rasheed and Yousafzai, 2015), since improvements in maternal mental health have been accompanied by improved interaction in prior studies (Rahman *et al*., 2013). Contrary to our expectations, there was a decline in maternal–child interaction after the intervention. The reasons for this finding are unclear but we offer several possible explanations. First, although the OMCI assessment tool we used was developed specifically for low-resource settings (Rasheed and Yousafzai, 2015), it has not been validated in Zimbabwe and may fail to capture important contextual elements of maternal–child interaction. Second, we utilised the same story book to conduct the baseline and endline assessment, to reduce variability between measurements, which may have resulted in children being less interested, or in mothers being unable to find new ways to interact with their child. Third, it is conceivable that women had less time to spend playing with their child because they were engaged in other activities such as CKT groups, and this was reflected in reduced interaction scores. Finally, it does not necessarily follow that reductions in maternal depression will lead to increases in mother–child interaction. We note similar findings from Rwanda, where some OMCI scores declined after a parenting intervention despite improvements in other measures such as early child development engagement and parental conflict (Barnhart *et al*., 2020).

Our study had strengths and limitations. This was a pilot study among a small group of women in one rural district of Zimbabwe, and findings cannot necessarily be generalised. Women were purposively sampled based on their prior involvement in the SHINE trial (Humphrey *et al*., 2019), and their previous research experiences may have shaped their reactions to the new intervention. There may have been courtesy bias in women's self-reported mental state at the end of the study, although results were consistent across all tools, and outcomes were measured by a separate cadre to those delivering the intervention. We also collected rich qualitative data, which strongly supported the quantitative findings. We did not have sufficient statistical power to evaluate child growth or development in this feasibility study, so we cannot comment on whether this intervention would impact child health; this should be evaluated in larger efficacy trials.

In summary, we show that problem-solving therapy can be delivered in rural communities by trained VHWs. Given the high prevalence of poverty and depression in rural sub-Saharan Africa (Lund *et al*., 2010), this study provides much-needed evidence to inform the scale-up of brief psychological interventions together with peer-support and income-generating activities, particularly in rural areas where the mental health treatment gap is most stark (Chibanda, 2017). There is now a need to ascertain how best to provide an integrated and sustainable community intervention, drawing on experiences from previous effective scale-up approaches (Chibanda, 2017). Given the adverse effects of poor maternal mental health on offspring (Lovejoy *et al*., 2000; Dennis and McQueen, 2009; Field, 2010; Surkan *et al*., 2011), future studies should evaluate the effects of this intervention on child health, growth and development.

## Supplementary material

For supplementary material accompanying this paper visit http://dx.doi.org/10.1017/gmh.2021.32. click here to view supplementary material
