# Supplementary material for: The Friendship Bench as a brief psychological intervention with peer support in rural Zimbabwean women: a mixed methods pilot evaluation
Source: Glob Ment Health (Camb). 2021 Aug 26;8:e31. doi: 10.1017/gmh.2021.32 (PMC8392686; doi:10.1017/gmh.2021.32)
Supplement: Supplementary file 1 [file S2054425121000327sup001.docx]

**Supplementary Table 1 – Baseline characteristics of enrolled versus non-enrolled women**

|  | **Enrolled** | **Not Enrolled** | **P-Value** |
| --- | --- | --- | --- |
|  |  |  |  |
| **Maternal factors^1^** | **N = 27** | **N = 4816** |  |
| Age, years; mean (SD) | 26.2 (6.7) | 28.4 (6.5) | 0.090 |
| Height, cm; mean (SD) | 161.0 (6.0) | 160.1 (5.9) | 0.466 |
| Married, % (N) | 92.6 (25) | 95.2 (4210) | 0.536 |
| Completed schooling, years; median (IQR) | 10 (7, 11) | 10 (9, 11) | 0.912 |
| Employed, % (N) | 0.0 (0) | 8.7 (379) | 0.116 |
| Religion, % (N) |  |  |  |
| Apostolic | 51.9 (14) | 47.2 (2134) | 0.093 |
| Other Christian | 29.6 (8) | 44.4 (2008) |  |
| Other religion | 18.5 (5) | 8.3 (377) |  |
| HIV-positive, % (N) | 29.6 (8) | 17.5 (832) | 0.099 |
|  |  |  |  |
| **Household factors^1^** |  |  |  |
| Household size; median (IQR) | 5 (3, 6) | 5 (3, 6) | 0.988 |
| Electricity, % (N) | 3.9 (1) | 2.7 (118) | 0.712 |
| Drinking water from improved source, % (N) | 69.2 (18) | 62.8 (2749) | 0.498 |
| Wealth quintile, % (N) |  |  |  |
| Lowest | 42.3 (11) | 19.7 (869) | 0.028 |
| Lower middle | 23.1 (6) | 19.9 (879) |  |
| Middle | 7.7 (2) | 20.3 (896) |  |
| Upper middle | 19.2 (5) | 20.1 (889) |  |
| Highest | 7.7 (2) | 20.1 (887) |  |
|  |  |  |  |
